# Modelling non-linear personality change surrounding transitions: A review of statistical approaches

**DOI:** 10.1177/08902070251376407

**Published:** 2025-09-08

**Authors:** Lisa Levelt, Joris Mulder, Nikki C. Lee, Maike Luhmann, Jaap J. A. Denissen

**Affiliations:** 1 Department of Developmental Psychology, 8125 Utrecht University, Utrecht, Netherlands; 2 Department of Methodology & Statistics, 7899 Tilburg University, Tilburg, Netherlands; 3 Faculty of Psychology, 9142 Ruhr University Bochum, Bochum, Germany; 4 German Center for Mental Health (DZPG), Bochum/Marburg, Germany

**Keywords:** nonlinear modeling, personality development, life events, longitudinal methods, machine learning

## Abstract

Personality changes surrounding transitions in life circumstances are often non-linear, presenting challenges for statistical analysis. This paper therefore reviews approaches to modelling non-linear personality change surrounding transitions, aiming to guide readers in selecting and applying an approach that fits their objectives. Seven approaches were reviewed, including traditional mixed-effects methods, continuous-time dynamic models, and relatively novel data-driven techniques. Each approach is explained, outlining its strengths and limitations. The approaches’ practical utility is assessed through a case study examining changes in life satisfaction surrounding widowhood, using LISS panel data. Interpretability and model fit are compared, and annotated R code is provided as a tutorial for implementation. Results highlighted the varied suitability of the mixed-effects approaches for studying different aspects of change. The data-driven techniques excelled in capturing average and person-specific trajectories, generalised effectively, and allowed interpretation of different change aspects than the mixed-effects approaches allowed for. Importantly, the approaches yielded distinct findings regarding life satisfaction changes surrounding widowhood, with theoretical implications. The paper concludes with practical recommendations for selecting and applying these approaches. By expanding the reader’s statistical toolkit and providing an accessible overview, this resource supports the effective analysis of non-linear changes surrounding transitions, enabling a fuller understanding of personality change.

## Plain language summary

Personality often changes surrounding major life transitions. For example, many people become more emotionally stable when starting their first job. Understanding these changes is important, because personality changes can influence important life outcomes, such as health and income. However, it is difficult to study these changes, because they are often not straightforward. Instead of steadily increasing or decreasing, personality trait levels may go up and down over time, with periods of faster or slower change. Common statistical methods are suited for studying straight-line changes (like steady increases), but if we use these methods to study non-straight-line personality change, we risk underestimating how much personality actually changes. Statistical methods to study non-straight-line change are less well-known, and it is unclear how to choose among alternatives and apply them. This paper reviews statistical methods to study non-straight-line change. We explain each method, discussing its strengths and weaknesses. We demonstrate how to apply the methods in a case study that examines how life satisfaction changes when people become widowed. The accompanying website illustrates how to conduct the methods in the free software environment R. We find that certain methods work well for quantifying specific aspects of change (such as how much and how quickly), while other methods are better for finding the overall pattern of change. Importantly, the methods led to different findings regarding life satisfaction changes surrounding widowhood, highlighting how the choice of method can impact research conclusions. The paper ends with practical recommendations for selecting and using the methods. Altogether, this paper equips readers with statistical tools to study non-straight-line change in order to gain further understanding of how personality changes surrounding transitions.

## Introduction

Personality traits are relatively consistent patterns in an individual’s thoughts, feelings, and behaviours, such as extraversion, self-esteem, and life satisfaction (Kandler et al., 2014). Changes in these traits predict important life outcomes − such as health, education, income, and marriage − beyond the predictive power of stable trait levels (Wright & Jackson, 2023). Theories emphasise that personality changes often occur in response to transitions (e.g. Dweck, 2017; Roberts, 2018; Wrzus, 2021). Transitions are discrete changes in life circumstances, such as graduating from school, entering a new relationship, or starting therapy. Transitions often involve shifts in routines, responsibilities, and roles, causing changes in thoughts, feelings, and behaviours, which may eventually produce personality trait changes (Bleidorn & Denissen, 2021). Some transitions bring similar changes in routines and responsibilities for many people, leading to systematic personality changes that are commonly experienced across individuals. Empirical evidence supports the notion of systematic personality change surrounding transitions, though the effects are generally modest (for a review, see Bühler et al., 2023). One reason for this may be that changes surrounding transitions tend to be non-linear (Bleidorn et al., 2020), which poses statistical challenges. A recent study found that modelling personality change with an unsuitable change shape can underestimate the extent of change by up to 64% (Wright & Jackson, 2024).

Personality change surrounding transitions often shows typical non-linearities. Specifically, change can be discontinuous, meaning that the personality trait level or its trajectory can shift abruptly (Luhmann et al., 2014; Roemer et al., 2024). For example, sudden increases in openness and agreeableness have been found surrounding the transition to retirement (Schwaba & Bleidorn, 2019). Furthermore, personality change surrounding transitions can be (partially) temporary, having a U-shaped form (Luhmann et al., 2014; Roemer et al., 2024). For instance, bereavement was found to be associated with large initial declines in life satisfaction, which recovered after five years (Asselmann & Specht, 2023). Discontinuous or temporary changes may be overlooked if analysed using linear models. Therefore, to study systematic personality change surrounding a transition, a statistical approach that appropriately captures non-linearity is crucial. The statistical literature on non-linear modelling is considerably more complex than that on linear modelling, however. This paper therefore reviews various approaches to the statistical modelling of non-linear personality change surrounding transitions, aiming to guide researchers in selecting and applying an approach that fits their research objectives.

### Considerations for modelling non-linear change

To study personality change, repeated measurements from the same individuals are needed. Analysing such multilevel data requires accounting for the nested structure of measurements within individuals. Moreover, as personality change can vary significantly between individuals (Haehner et al., 2024a, 2024b; Schwaba & Bleidorn, 2018; Wright & Jackson, 2024), analytic methods should account for potential individual-specific trajectories.

When examining non-linear change, it is important to use the precise timing of measurements (Sciarra, 2023). For transitions, the focus is on the timing of measurements relative to the transition occurrence. Because many transitions occur infrequently, large-scale panel studies are often used to achieve sufficient statistical power to study personality change surrounding these events (Haehner and Krämer et al., 2025). Such studies typically use fixed measurement schedules, but as participants experience transitions at different points during the study, the timing of measurements relative to the transition varies across individuals. For example, personality measurements may occur annually in the same month for all participants, but Person A’s first post-transition measurement might be 2 months after the transition, while for Person B it is 9 months after the transition. Using these precise timings in analyses is essential for valid insights into how changes unfold over time (McCormick et al., 2023). Therefore, an effective statistical approach should accommodate distinct measurement timings relative to the transition across individuals.

Consequently, a statistical approach to studying personality change surrounding transitions should account for non-linearity, individual differences, and distinct measurement timings relative to the transition across individuals. Mixed-effects models (also called random coefficient or multilevel models) are well-suited for this, as they allow researchers to specify a specific non-linear function for the average trend while also accounting for individual deviations around this average. Continuous-time dynamic models and certain more data-driven techniques offer further flexibility to capture non-linear patterns and individual differences therein. All these approaches can handle distinct measurement timings relative to the transition across individuals.

Other models can also be used to study personality change surrounding transitions, such as latent growth curve models within the discrete-time structural equation modelling (SEM) framework (e.g. Hutteman et al., 2015), and fixed-effects models (e.g. Krämer et al., 2025). However, to describe population-level change using these approaches, they require consistent measurement timings relative to the transition across individuals. For example, both Person A and Person B’s first post-transition measurement must occur the same number of months after their transition, or their measurements must be grouped into broader categories, such as a ‘0–12 months’-category. This categorisation reduces the precision of measurement timings. Consequently, this paper does not focus on discrete-time SEM or fixed-effects models. However, a comparison between these approaches and those included in the current study will be revisited in the discussion. In addition, many of the mixed-effects models examined here can also be estimated within the discrete-time SEM framework, which will also be addressed in the discussion.

### Selected modelling approaches

In this paper, we examine seven approaches to statistical modelling of non-linear personality change surrounding transitions. These approaches were chosen either because they are commonly used to study personality change or because of their potential value in studying this change. Specifically, we considered:

• two approaches that approximate the overall shape of change with a single researcher-specified function for the personality change: linear regression on a transformed time variable and non-linear regression;
• two approaches that approximate the overall shape of change using multiple researcher-specified functions fit to portions of the overall trajectory of personality change: piecewise regression and changepoint analysis;
• one approach that approximates the overall shape of change with a researcher-specified function for the rate of change rather than the personality trait level directly: continuous-time dynamic models;
• and two data-driven approaches that freely estimate the shape of change in the trait level: generalised additive models and Gaussian process regression.

The approaches differ in several key aspects. First, they vary in their flexibility to capture different shapes of changes. Some are more suited to capturing discontinuous (instantaneous) versus continuous (gradual) patterns. They also differ in the number of modelling choices that the researcher is required to make, and the sensitivity to these choices. In terms of their interpretability, the different approaches use different types of parameters, which provide insights into different aspects of change, such as the extent, rate, or timing. Another important difference is the degree to which models risk overfitting to the sample data, which can limit the replicability and generalisability of findings. Additionally, the approaches differ in their ability to account for individual differences and to incorporate moderators to explain these differences. Finally, the approaches vary in terms of data requirements, software implementation, and computational efficiency. Given these differences, it is important to examine when and how to use each approach. This paper reviews all these considerations.

### The present paper

The first part of this paper describes each approach and highlights their advantages and disadvantages. The second part demonstrates the practical utility of the approaches through an empirical application: the change in life satisfaction surrounding the transition to widowhood, using LISS panel data (Scherpenzeel & Das, 2011). This example was chosen because prior research has identified systematic non-linear changes in life satisfaction surrounding widowhood, characterised by both discontinuous (Asselmann et al., 2021; Denissen et al., 2019), and temporary features (Asselmann & Specht, 2023; Doré & Bolger, 2018). This makes it a suitable test case for evaluating the approaches’ interpretability, and their performance in capturing typical patterns of personality change surrounding transitions. It is worth noting that the analyses were not preregistered, as they were initially conducted for learning purposes rather than formal comparison. We will come back to this point in the discussion. For transparency, we have created an accompanying website with annotated R code (https://lisalevelt.nl/publications/modelling-nonlinearity/tutorial/), detailing the modelling steps and decisions per approach. This resource also serves as a tutorial for implementing the approaches.

To our knowledge, no previous review has examined approaches for multilevel modelling of non-linear change surrounding transitions specifically. Other resources on modelling non-linear change (e.g. Arin et al., 2022; Cudeck & Harring, 2007) did not focus on multilevel data, or did not compare approaches from the mixed-effects framework to continuous-time dynamic models and relatively novel data-driven techniques. Given that these newer methods have been highlighted for their potential for personality research (Bleidorn & Hopwood, 2019; Stachl et al., 2020) and longitudinal data analysis (Sheetal et al., 2023), it is important to evaluate them alongside more traditional approaches. Overall, the findings will contribute to establishing best practices for analysing personality change surrounding transitions.

## Approaches to statistical modelling of non-linear change surrounding transitions

This section explains seven approaches to statistical modelling of non-linear personality change surrounding transitions. Note that, for all approaches, we considered time as coded relative to the transition (e.g. the timing of measurements in months before and after widowhood). While the models are discussed in the context of having measurements both before and after the transition, they can also be applied when only pre- or post-transition data are available. We included mathematical equations to explicitly specify the function each approach uses to capture temporal changes. Since our primary focus is on modelling systematic change, the equations depict only the fixed-effects component, with error terms omitted for simplicity. We also discuss for which parameters software supports the incorporation of random effects.

### Linear regression on a transformed time variable

To study personality changes surrounding a transition, personality trait levels are typically regressed on a variable indicating time relative to the transition. For a straight-line change trajectory, this involves regressing the trait level directly on the time variable. A first approach to capture non-straight-line trajectories, however, is to regress personality trait levels on a transformation of the time variable instead.

**Figure 1.**
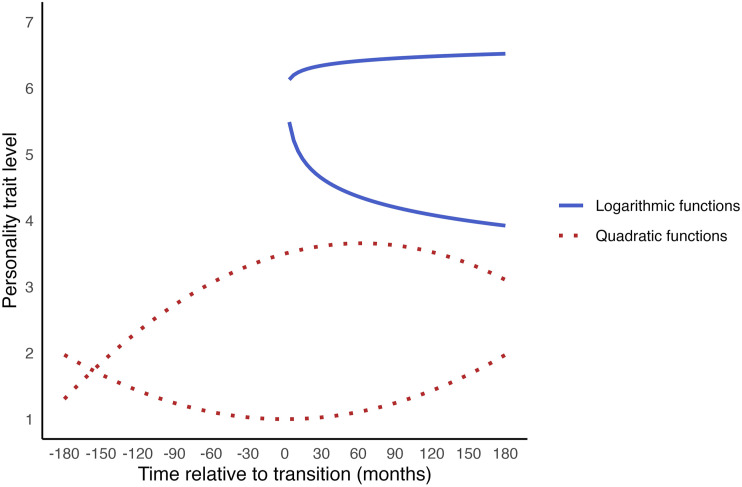
Examples of change trajectories of logarithmic and quadratic functions.

One example of such a transformation that has previously been used to model personality changes surrounding transitions is a logarithmic transformation (e.g. Mangelsdorf et al., 2019). A logarithmic transformation of time can be used to capture patterns where changes are more pronounced near the transition and gradually level off over time. This approach has shown, for instance, that self-esteem increased after major life events, most notably in the first four years following the event, after which it gradually stabilised (Mangelsdorf et al., 2019). Note, however, that a logarithmic transformation can only be used to analyse post-transition data, as we cannot take the logarithm of negative values. Another example is a quadratic transformation, which can also be used to analyse pre-transition data and can capture a U-shaped pattern of change surrounding the transition. Research using this transformation has demonstrated an inverted U-shaped pattern of change in neuroticism surrounding the start of military service, with an initial increase followed by a decrease (Magal et al., 2021). Figure 1 illustrates examples of change trajectories that can be captured using quadratic and logarithmic functions.

Various other transformations are possible; each involves adding the transformation of time to a linear regression model. Since this transformation enters additively − just like any other predictor − this approach is technically still a linear regression model (Cudeck & Du Toit, 2002). Therefore, we refer to this approach as ‘linear regression on a transformed time variable’. As the transformation is simply added to a linear regression model, this approach is straightforward to implement.

To illustrate this approach, consider a quadratic transformation. By adding a quadratic term of time to the linear model, a second slope alongside the initial (linear) slope is estimated:

(1) $$\text{Trait}\;\text{level}=intercept+slope1\times \text{Time}+slope2\times {\text{Time}}^{2}$$

In this equation, the first slope, denoted by *slope*1, controls the direction and rate of change near the transition, while the second slope, *slope*2, determines the curvature, bending the trajectory upward or downward as the timing becomes more distant (in both directions) from the transition, creating a U-shaped pattern of average personality change over time. Together, these coefficients determine the timing of the peak or valley of the U-shape. We can identify this timing by calculating:

(2) $$\text{Time}\;\text{of}\;\min\;/\;\max\;\text{trait}\;\text{level}=-\frac{slope2}{2\times slope1}$$

This equation illustrates how a transformation of time can model non-straight-line changes while generating interpretable parameters that describe the change in an understandable shape.

Beyond quadratic functions, we can use various other transformations to model different shapes of change. Examples include square-root transformations of time and higher-order polynomials such as cubic functions. This approach is further easily extended to include random intercepts and slopes to account for individual differences in trajectories. Moreover, interactions between moderator variables and slopes can be used to explain differences in these individual trajectories.

This approach is appealing for its simplicity, but also has limitations. The estimated change can only follow very specific shapes implied by the chosen transformations. If the selected transformations are inaccurate, this approach can lead to misguided conclusions. Additionally, the range of shapes that can be modelled using mathematical transformations of time is limited, making it difficult to capture complex trajectories. Higher-order polynomials can approximate complex curves, but they require numerous time points. Another limitation is the inability to model plateaus. For instance, while a logarithmic transformation can suggest when change becomes minimal, a quadratic transformation indicates only the peak or valley, not when trends start or stop. Moreover, as these functions spread their trends across the entire time span, their curvature may be disproportionately influenced by observations at the edges of the timeframe (Fjell et al., 2010). Nevertheless, when the expected shape of change aligns with a simple transformation of time, this approach offers easy implementation and interpretation.

### Non-linear regression

Linear regression has an additive form, where personality trait levels are predicted by a sum of predictors, possibly including transformations of time. We can also move beyond this additive form by using a mathematical formula for average personality change where parameters relate in non-additive ways − such as through division or exponentiation. These functions are non-linear, hence the term ‘non-linear regression’. Non-linear functions offer greater flexibility than linear functions in modelling diverse shapes of change, including trajectories with plateaus (Miguez et al., 2018). An example of non-linear regression in the study of personality change surrounding transitions is its use in showing that major life events are associated with changes in life satisfaction, some of which are long-lasting (Anusic et al., 2014; Yap et al., 2012).

To illustrate non-linear regression, let us consider the following pattern of change: Long before the transition, the personality trait level is relatively stable. Near the transition, it temporarily increases or decreases. Afterwards, it gradually returns to its initial level and stabilises again. To model this pattern, we need a mathematical function that approximates this shape. One such function is a bell curve or Gaussian density function^1^:

(3) $$\text{Trait}\;\text{level}=baseline+amplitude\times \exp\left(-\frac{{\left(\text{Time}-centre\right)}^{2}}{2\times {width}^{2}}\right)$$

**Figure 2.**
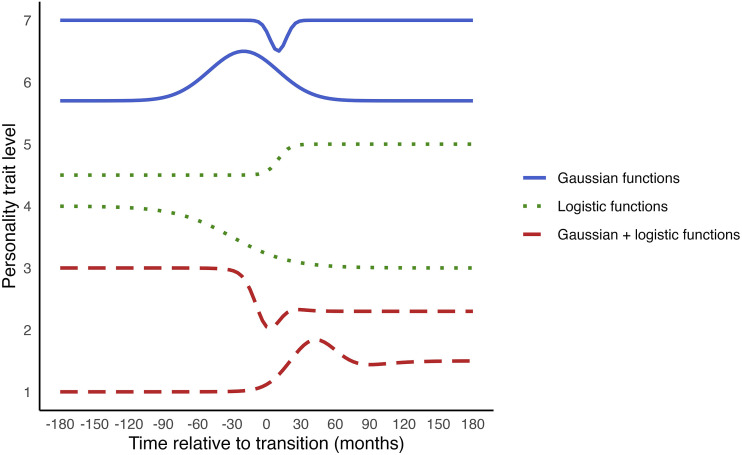
Examples of change trajectories of non-linear functions.

In this function, the *baseline* parameter represents the initial level of the personality trait; the *amplitude* indicates the height of the peak or valley of the trajectory; the *centre* indicates the timing of the peak/valley; and the *width* controls how quickly the personality trait level changes from baseline towards, and away from, the peak/valley. We can use the value of *width* to calculate the time span over which the personality trait level changes, which we will demonstrate in the example application. These parameters illustrate that non-linear regression provides interpretable values describing meaningful aspects of change. It is important to note that when using a Gaussian function, we only model temporary change, as this function dictates that the personality trait ultimately returns to its pre-transition stable level after a temporary increase or decrease. The blue solid lines in Figure 2 show examples of change trajectories that can be captured using Gaussian functions.

We might expect another pattern of personality change surrounding transitions: an initial stable phase, followed by a gradual shift to a new level during the transition, where it then stabilises again. In this case, there is lasting change. Such a pattern can be modelled using sigmoidal functions, such as the logistic curve^2^:

(4) $$\text{Trait}\;\text{level}=step\;size\times \left(\frac{1}{1+\exp\left(-\frac{\left(\text{Time}-centre\right)}{width}\right)\;}\right)$$

Here, the *step size* parameter (because the function gradually ‘steps’ to a new stable level) indicates the difference between the initial level and the new stable level. The *centre* indicates the timing at which half of the step is reached; and the *width* controls how quickly the personality trait level changes from baseline to its new stable level. The green dotted lines in Figure 2 show example change trajectories that logistic functions can capture.

These functions can also be combined to model more complex patterns of change. For example, a function that adds the logistic function to the Gaussian density function can model a pattern of an initial stable period, a temporary peak or valley, and a gradual shift to a new stable level. The red dashed lines in Figure 2 show examples of such change trajectories. This illustrates how non-linear regression provides flexibility for modelling various shapes of change. These functions are relatively straightforward to implement. Instead of a linear regression equation, we use the non-linear equation that reflects our expected shape of change in the model formulation.

A few limitations of non-linear regression should be noted. In theory, it is possible to add random effects for all parameters to account for individual trajectories. However, in practice, when using complex functions with many parameters, random effects can only be added for a limited number of parameters. Otherwise, convergence issues may arise, as we will also encounter in the example application. Similarly, estimating interactions between parameters and moderator variables will require large samples. Unfortunately, little guidance exists on how large these samples must be. Required size likely depends heavily on the complexity of the non-linear function. Yap and Colleagues (2012) found it infeasible to include moderators in their custom non-linear function, despite having 562–1742 participants with an average of eight observations each. Yan and Su (2006) have proposed formulas for determining sample sizes in non-linear mixed-effects models with interactions, where the exposure group shows non-linear change and the control group linear change. They suggest smaller sample sizes may sometimes suffice. A final limitation of non-linear regression is that, similar to linear regression using a transformed time variable, it requires researchers to specify the expected shape of change in advance. If this specification is inaccurate, this can lead to misguided conclusions. Despite these limitations, non-linear regression offers high flexibility and interpretability.

### Piecewise regression

So far, we have considered approaches in which the researcher defines one overall shape of change. However, changes surrounding transitions may be discontinuous, meaning that the level of the personality trait or its trajectory shifts abruptly. We can capture these discontinuous features by fitting separate functions to different portions of the observational period – this is known as piecewise, multiphase or segmented regression (Cudeck & Klebe, 2002). For example, different segments could model different trajectories before and after the transition, with possible abrupt level changes at the time of the transition. Research using such a model has shown, for instance, that women’s self-esteem increases before the transition to parenthood, drops sharply around childbirth, and then gradually decreases over the following years (Bleidorn et al., 2016). Such a change pattern does not fit standard mathematical functions like a bell curve or sigmoid, but piecewise regression allows for modelling these custom change patterns.

**Figure 3.**
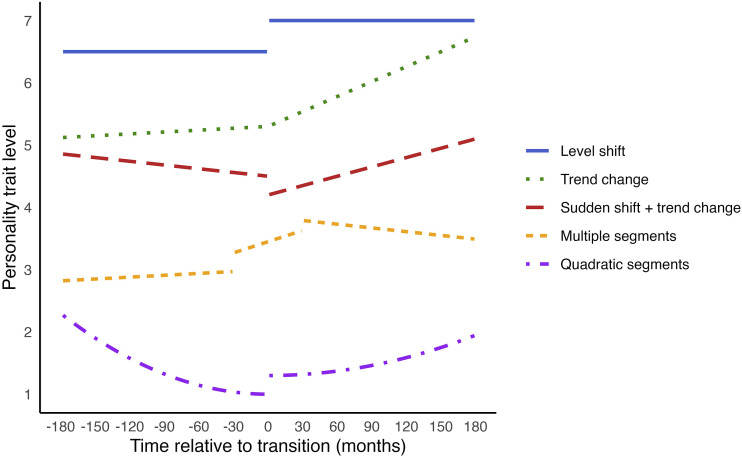
Examples of change trajectories of piecewise functions. *Note.* Changepoint analysis (discussed in the next section) can capture similar shapes of change.

To understand piecewise regression, consider its simplest case: using one linear segment to model the period before the transition and another segment for the period after. This can be implemented by creating variables to quantify the changes pre- and post-transition, as for example done by Luhmann and Eid (2013). One change we might expect is in the overall personality trait level, or intercept. To measure the shift in the intercept after the transition, we can use a dummy variable (postD), coding it as 0 for all measurements before the transition and 1 for all measurements after. The parameter for this variable will quantify the shift in the personality trait level after the transition, while the original intercept captures the level before the transition. The blue solid line in Figure 3 shows an example of a change trajectory with a personality trait level shift.

We may also expect different trends pre- and post-transition. To model these, we create two new time variables: one (preLin) for the period before the transition, which has negative values indicating the time before the transition, and is 0 after the transition; and another (postLin) for the period after the transition, which is 0 before the transition and has positive values afterwards indicating the time after the transition. These variables capture the rates of change pre- and post-transition, respectively. The green dotted line in Figure 3 shows an example of a trajectory with a trend change.

When modelling both sudden shifts and trend changes (as in Figure 3, red long-dashed line), the fixed-effects equation becomes:

(5) $$\begin{array}{l} \text{Trait}\;\text{level}=pretransition\;intercept+posttransition\;intercept\;change\times \text{postD}\\ +pretransition\;slope\times \text{preLin}+posttransition\;slope\times \text{postLin}\; \end{array}$$

By combining parameters that apply to specific periods, custom shapes of change can be modelled. Even when modelling complex change shapes, the parameters remain interpretable.

The two-piece linear-linear model can be extended in several ways. First, random effects can be included for each parameter to account for individual differences in these change aspects. Additionally, interactions with moderator variables can be tested for any of the parameters. Furthermore, more segments can be used (as in Figure 3, orange short-dashed line), and each segment can be modelled using different change shapes, such as using quadratic functions (as in Figure 3, purple dash-dotted line).

The potential for adding segments with specific shapes makes piecewise regression flexible, but also introduces many choices to be made by the researcher: how many segments to include in the equation; what time periods should the segments cover; and what mathematical functions to use for the segments? All these choices affect the results, and can lead to misguided conclusions if made inaccurately (Doré & Bolger, 2018). For example, using too few segments could obscure fluctuations within segments. This issue can be addressed by comparing the fit of different segment configurations (cf., Bleidorn et al., 2016). However, limited time points often restrict analyses to models with only two or three linear segments, which are unlikely to be realistic depictions of change. Furthermore, researchers must decide the beginning and end of each segment, that is, where the trajectory is expected to shift, as testing every possible time point would be impractical. Because this point is fixed in piecewise regression, it is assumed that the trajectory shifts at the same time point for all individuals, which is likely unrealistic. Despite these drawbacks, piecewise regression could strike a good balance between flexibility and interpretability.

### Changepoint analysis

As stated above, an important consideration in piecewise regression is deciding the beginning and end of the segments. In fact, identifying when the trajectory changes surrounding a transition, the so-called changepoint, may be of key interest. For instance, we might want to know if and when certain changes emerge already in anticipation of a transition, or when personality stabilises again after an initial change in response to a transition. Questions such as these can be addressed using changepoint analysis, in which a parameter is added to piecewise regression to estimate when the trajectory changes course (Cudeck & Klebe, 2002; Preacher & Hancock, 2015). For instance, Martin and colleagues (2014) used this approach to study how the timing of entry into work, marriage, and parenthood influenced the development of antisocial behaviour during young adulthood.

To explain changepoint analysis, we will consider it within the standard linear framework, which allows use of standard regression parameters, though other approaches exist (Breit et al., 2023). Muggeo’s et al. (2014) approach is widely used in this context (Breit et al., 2023). In this approach, a changepoint parameter is incorporated into a reparametrized version of the piecewise linear regression equation discussed in the previous section. This reparameterization avoids creating separate time variables for the segments, as the timing of these segments is unknown. Rather than estimating a separate slope for the second segment, the reparametrized model estimates the change in slope that occurs after the changepoint. The location of the changepoint is a parameter that is estimated. The model uses an indicator function ‘I(Time > changepoint)’ that equals 1 if time is greater than the changepoint and 0 otherwise. The model is:

(6) $$\begin{array}{l} \text{Trait}\;\text{level}=intercept+initial\;slope\times \text{Time}\;\\ +slope\;change\;after\;changepoint\times I\left(\text{Time}>changepoint\right) \end{array}$$

In this model, the *initial slope* indicates the trend before the changepoint. The sum of the *initial slope* and the *slope change* indicates the trend after the changepoint. The reparametrized piecewise regression model in changepoint analysis preserves the advantages of being able to model custom shapes of change (for example, see Figure 3), while parameters remain interpretable.

An additional advantage is that, since the timing of the changepoint is estimated as a parameter, the uncertainty around this estimate is also quantified given the observed data. Even with a theoretical basis for pre-specifying the changepoint’s location, as in regular piecewise regression, failing to account for the uncertainty in this location can affect the slope estimates, and result in underestimation of standard errors, leading to inflated confidence in the effects. While the standard errors in changepoint analyses may be larger, they more accurately reflect statistical uncertainty, potentially resulting in more meaningful and reliable piecewise regression parameters.

Moreover, treating the changepoint as a parameter enables the inclusion of individual differences in this point. Estimating random effects for the changepoint accounts for variations in the timing of shifts in trajectories surrounding a transition. This is another advantage compared to the traditional piecewise regression specification, which assumes a uniform changepoint across all individuals − an assumption that is likely unrealistic. Additionally, moderators can be added for the changepoint’s location to explore factors that explain why some individuals experience earlier or later shifts in their trajectories.

Despite its potential, application of changepoint analysis is somewhat limited by current software implementations. To our knowledge, there are two R packages that support changepoint analysis with random effects. Each has limitations, however. The ‘segmented’ package (Muggeo, 2024), which relies on frequentist estimation, is restricted to estimating only one changepoint (i.e. trajectories with two segments). Furthermore, it can only quantify slope differences between segments, but not sudden shifts in the personality trait level (it estimates one overall intercept that applies across the trajectory). It does allow for random effects in the changepoint’s location, the intercept, and the slopes. In practice, however, it is often necessary to restrict the correlation between random effects to zero for the model to converge, in which case dependencies among them are missed. Alternatively, the ‘mcp’ package (Lindeløv, 2024), which relies on Bayesian estimation, was developed specifically to detect multiple changepoints in a trajectory and supports random effects for these changepoints. However, it does not support random effects for intercepts and slopes. Both packages are limited to estimating straight-line segments. Thus, while changepoint analysis holds promise, further software development is needed.^3^

### Continuous-time dynamic models

The approaches discussed so far directly model personality trait levels over time. Continuous-time dynamic models (CTDMs; Voelkle et al., 2012) adopt an alternative approach by estimating how the *rate of change* of the personality trait levels, that is, the derivative, evolves over time. Using a linear function for the rate of change results in non-linear changes in personality trait levels. For instance, the rate of change might be faster closer to a transition, but linearly slow down further from it. Although the rate of change decreases linearly, the resulting trait trajectory is non-linear. The linear model for the rate of change yields interpretable parameters describing how the trait level changes over time. An example of CTDM’s application to study personality change surrounding transitions is a study which showed that self-esteem temporarily decreased after negative events (Haehner and Driver et al., 2025).

In CTDM, personality trait level changes are modelled with a set of two differential equations, which estimate the rate of change (the derivative) at a given time point (Driver & Voelkle, 2018b):

(7) $$\begin{array}{l} \text{Trait}\;\text{level}\;\text{change}\;\text{rate}\;\text{at}\;\text{Time}\;t=\text{trait}\;\text{level}\;\text{at}\;\text{Time}\;t\\ \times trait\;auto\;effect\\ +\text{transition}\;\text{response}\;\text{level}\;\text{change}\;\text{rate}\;\text{at}\;\text{Time}\;t\; \end{array}$$

(8) $$\begin{array}{l} \text{Transition}\;\text{response}\;\text{level}\;\text{change}\;\text{rate}\;\text{at}\;\text{Time}\;t=\\ transition\;input\times \text{transitionTime}\\ +t\text{ransition}\;\text{response}\;\text{level}\;\text{at}\;\text{Time}\;t\times transition\;auto\;effect\; \end{array}$$

Additionally, the personality trait level at the first time point is estimated as the *initial level.*

Equation (7) estimates the rate of change in the trait level at a given moment, based on the trait’s level at that moment, and the parameters describing the change dynamics − the rest of the equation to which we will turn later. However, generally we are interested in estimating change over a period of time (e.g. over the first post-transition month or year), rather than at a single time point. To estimate the change over a time period, we can replace ‘trait level at Time t’ with the trait level at the previous time point (or the *initial level* for the first time point), along with the time period over which change is estimated. In this way, CTDM describes change as a function of the time period over which it is estimated, which is a key strength: this produces change estimates that allow comparisons across studies using different timescales. For example, the earlier-mentioned study on self-esteem ((Haehner and Driver et al., 2025) applied CTDM to data on daily, monthly, and yearly timescales and found temporary decreases in self-esteem when measured daily or monthly, but not when measured yearly. This illustrates how CTDM can reveal how change processes may differ depending on the timescale.

We now turn to the parameters describing the change dynamics in equation (7). Over time, the trait level fluctuates randomly, as is captured by the model error (which is omitted from all model equations for simplicity). The *trait auto effect* describes how the trait responds to these fluctuations. A negative *auto effect* means that after a fluctuation, the trait tends to return to its *initial level*. A positive *auto effect* indicates that a fluctuation leads to further divergence from the *initial level*. A near-zero value means that fluctuations persist over time. The final term in equation (7), *transition response level change rate at Time t,* captures how the trait responds to the transition.

**Figure 4.**
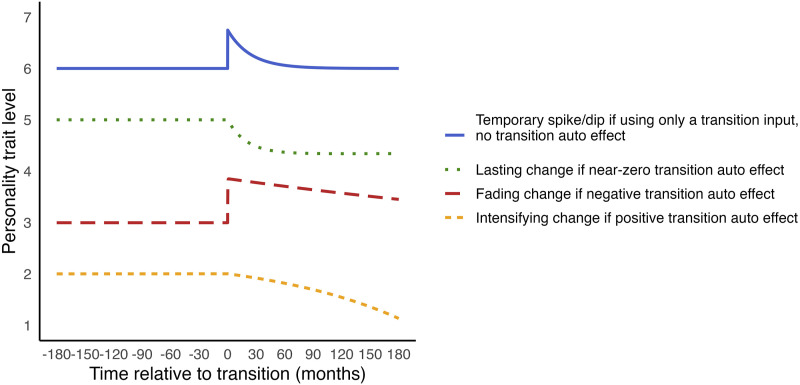
Examples of change trajectories of continuous-time dynamic models.

The transition is modelled as an input that disrupts the usual evolution of the personality trait (Driver & Voelkle, 2018b). How the response of the trait to the transition evolves over time is captured by equation (8). The input can cause a sudden spike or dip in the trait level. To capture the timing of this input, we need to create a time variable (transitionTime) that is zero except at the timing of the transition, where it is set to one. The magnitude of the spike or dip is estimated by the *transition input* parameter. Although the input causes an immediate change, whether this change persists depends on the *auto effects*. For example, with a negative *trait auto effect*, the trait will return to its *initial level* over time, resulting in a temporary change, as in the blue solid line in Figure 4.

Sustained change after the transition can be captured by the *transition auto effect.* If this parameter is constrained to zero, the transition input initiates a stable process that results in sustained change, as illustrated by the green dotted line in Figure 4. When the *transition auto effect* is estimated freely, the extent to which the transition effect is temporary or lasting is estimated. A negative *transition auto effect* indicates fading change (Figure 4, red long-dashed line); a near-zero *auto effect* indicates lasting change (Figure 4, green dotted line); and a positive *auto effect* means intensifying change (Figure 4, orange short-dashed line). Thus, CTDM can capture temporary or sustained changes, or a combination of both (Driver & Voelkle, 2018b). A final note on CTDM’s parameters is that they interact. Their absolute values are not directly interpretable, and their combined effects are best understood through visualisation.

CTDMs with random effects can be estimated using the ‘ctsem’ R package (Driver et al., 2017). This package supports both frequentist and Bayesian estimation. The frequentist approach is faster but allows only random intercepts. The Bayesian approach allows random effects for all parameters, but is more computationally demanding (Driver & Voelkle, 2018a). In our empirical example (∼200 individuals with ∼11 observations each), estimation took only a few minutes, but studies with larger datasets (Haehner and Driver et al., 2025 included on average ∼1000 individuals with ∼5 observations each in their models) have reported computation times exceeding a week. The ctsem package also supports testing of moderator effects. As it is built on the structural equation modelling framework, it allows for the inclusion of measurement models that link observed indicators to latent variables. This enables distinguishing model error due to measurement error.

Two drawbacks of CTDM should be noted. First, its application to studying transitions has so far focused on modelling them as occurring at a known, instantaneous time point, without accounting for potential anticipatory effects (Driver & Voelkle, 2018b). Second, implementing CTDM in ctsem requires specifying parts of the model’s differential equations, which are less familiar in the psychology literature. Although examples are available (e.g. Driver, 2024; Driver & Voelkle, 2018b), tailoring the model requires some understanding of the mathematical logic, which can be challenging. Nonetheless, CTDM offers a flexible framework for studying the role of time in personality change surrounding transitions.

### Generalised additive models

The previously discussed approaches require researchers to specify many assumptions about the expected changes. In contrast, generalised additive models (GAMs; Hastie & Tibshirani, 1986) offer a data-driven approach for estimating trajectories surrounding transitions. GAMs estimate the overall shape of change from the data, using a sum of gradually connected functions, allowing them to flexibly capture any gradual change pattern, including periods of increases, decreases, and plateaus. An example of how GAMs have been useful to study personality trait changes surrounding transitions is that research using GAMs found that life satisfaction declines around widowhood, and only partially recovers afterwards (Doré & Bolger, 2018). This finding contrasts with studies using more rigid piecewise regression models, which reported full recovery (Asselmann & Specht, 2023).

**Figure 5.**
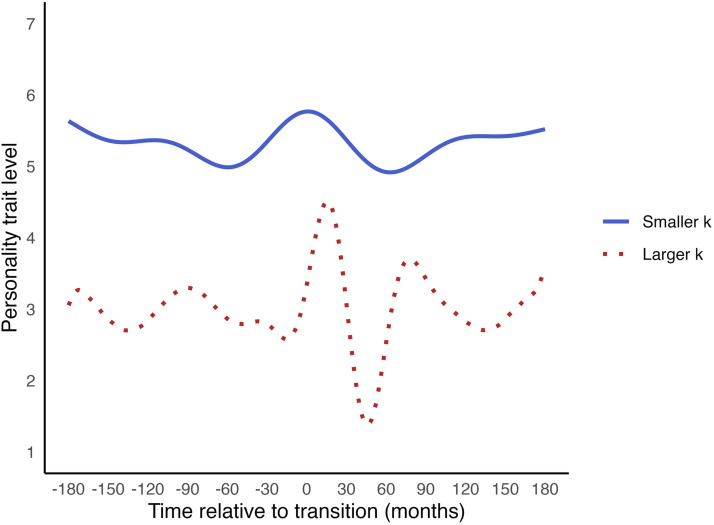
Examples of change trajectories of generalised additive models.

In GAMs, a smooth function is estimated to describe how a personality trait changes over time. This smooth function is typically a spline, which is a piecewise continuous curve. It is piecewise because it consists of multiple ‘subfunctions’, each modelling change over a specific time range. It is continuous because these subfunctions are connected seamlessly. Figure 5 shows examples of change trajectories that generalised additive models can capture using splines. Various types of splines exist, a common type being the cubic spline. A cubic spline estimates the optimal combination of cubic (third-degree polynomial) functions, with constraints ensuring they connect seamlessly. Specifically, these constraints are that the first and second derivatives of the functions must be equal at the points where they join. By using smooth functions that combine multiple subfunctions, GAMs can closely fit complex change patterns.

Fitting the data too closely, however, risks capturing random noise. This can reduce the model’s ability to generalise to new data. To counter this, GAMs automatically optimise the smoothness of the model. Before estimation, an upper limit for the model’s complexity, or ‘wiggliness’ is set, denoted as *k*. The choice of *k* is not critical; it should be large enough to represent the underlying pattern but small enough to maintain computational efficiency (Wood, 2017). A guideline could be setting *k* to one fewer than the number of unique time points, allowing a potential wiggle (curvature) at each point (see also Doré & Bolger, 2018). During model fitting, the complexity of the smooth function is reduced through penalisation, which shrinks the coefficients of subfunctions that contribute little to model fit. This eliminates unnecessary fluctuations that do not significantly improve the model (Wood, 2011). The result is the ‘effective degrees of freedom’ (EDF), which reflect the degree of non-linearity in the change after penalisation. The EDF ranges from 1 to *k*, where 1 indicates that the effect of time is approximately linear (no curvatures significantly improved model fit), and larger EDF values suggest greater non-linearity.

The smooth function is tested for statistical significance as in standard regression: assessing whether it differs from zero. However, *p*-values for smooth functions do not account for uncertainty in the optimal degree of penalisation. We therefore recommend treating these *p-*values as suggestive rather than definitive evidence for change, and advise to visually inspect the smooth function – assessing whether it displays a clear, interpretable pattern, and whether its confidence intervals are narrow and exclude a flat line over any meaningful time periods.

Further insight can be gained by examining slope changes across the range of time. Time periods where the confidence interval of the slope straddles zero indicate periods of stability. Positive slopes indicate increases, while negative slopes indicate declines. Again, note that these confidence intervals are not corrected for penalisation uncertainty, and the smoothing process in GAMs can spread out changes that may, in reality, be more sudden or drastic. Still, slopes across time points can provide a rough indication of when and how change is taking place (cf., Tervo-Clemmens et al., 2023). We will demonstrate this in the example application.

In GAMs, we can also examine individual differences in change trajectories and study whether moderators explain these differences. To account for individual differences, random effects can be added for intercepts (baseline levels), slopes (the overall linear trend through the wiggles), and shape of wiggles. The latter allows for person-specific non-linear curves that follow their own pattern, but share the same number of wiggles (Wood, 2017). Additionally, we can investigate whether different levels of a moderator predict distinct (non-linear) trajectories over time.

Some readers might wonder why we focus on GAM, a broader framework that typically uses splines to estimate smooth functions, instead of discussing (smoothing) splines as standalone models (e.g. Perperoglou et al., 2019), which is also possible. The main reason is that GAMs make it easier to include covariates with potentially linear effects alongside the non-linear effect of time. These covariates can be incorporated into the model as in any multiple regression model. This enables us to examine the non-linear effect of time, and each of the covariates’ effects, while keeping all other variables constant. Additionally, the 'gratia' R package (Simpson & Singmann, 2024) provides functions for obtaining the estimated slopes across time points for GAMs. These are more difficult to obtain for standalone splines.

Two disadvantages of GAMs should be noted, however. First, although GAMs optimise smoothness, they can be prone to overfitting, which reduces the generalisability of the results. A solution is to use cross-validation: splitting the data for model estimation and validation to test how well the findings generalise to independent data. Second, GAMs do not provide easily interpretable parameter estimates for the extent and rate of change. Still, if the goal is to flexibly model non-linear change in a data-driven way, GAMs are relatively easy to implement and provide valuable insights into complex patterns of change.

### Gaussian process regression

Another flexible approach that does not require pre-specifying a shape of change but estimates this shape data-driven is Gaussian process regression, a technique from the Bayesian machine learning literature (GPR; Rasmussen & Williams, 2006). GPR has previously been used to analyse longitudinal panel data, such as in research investigating how individuals’ preferences for submitting to authorities change over time (Karch et al., 2020). Although it has not yet been widely applied to study changes surrounding transitions, its flexibility makes it well-suited for this purpose. GPR can estimate any continuous (gradual) shape of change and provides parameters that describe the rate, extent, and timing of change.

GPR models non-linear change using ‘kernels’. Kernels are covariance functions, which take two inputs (time points) and return the covariance between the outputs (personality trait levels) at those inputs. This covariance quantifies how (dis)similar the outputs are, and thus how wiggly or smooth the trajectory is. The most common covariance function for modelling smooth, continuous trajectories is the exponentiated quadratic (EQ) kernel, defined as:

(9) $$\begin{array}{l} \text{Covariance}\;\text{of}\;{\text{trait}\;\text{level}\;\text{at}\;\text{Time}}_{a}\;\text{with}\;\text{trait}\;\text{level}\;\text{at}\;{\text{Time}}_{b}\\ ={magnitude}^{2}\times \exp\left(-\frac{{\left({\text{Time}}_{a}-{\text{Time}}_{b}\right)}^{2}}{{2\times length\;scale}^{2}}\right)\; \end{array}$$

**Figure 6.**
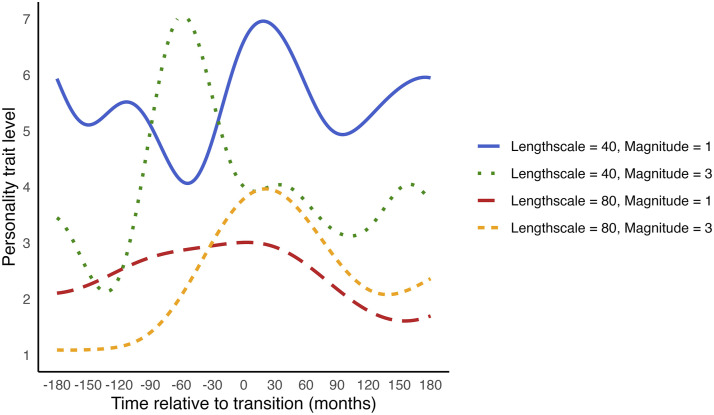
Example change trajectories of EQ kernels with different magnitudes and length scales.

The EQ kernel models changes in personality trait levels over time by reducing covariance (similarity) between personality trait levels as time points become more distant. The parameters − *length scale* and *magnitude* − govern the rate and extent of change, respectively. The length scale determines the rate of change: a smaller length scale indicates that similarity decreases rapidly as time points move apart, reflecting faster changes. The magnitude controls the extent of change, with larger values indicating greater variance from the mean personality trait level, or larger changes. Figure 6 shows example trajectories with varying magnitudes and length scales.

**Figure 7.**
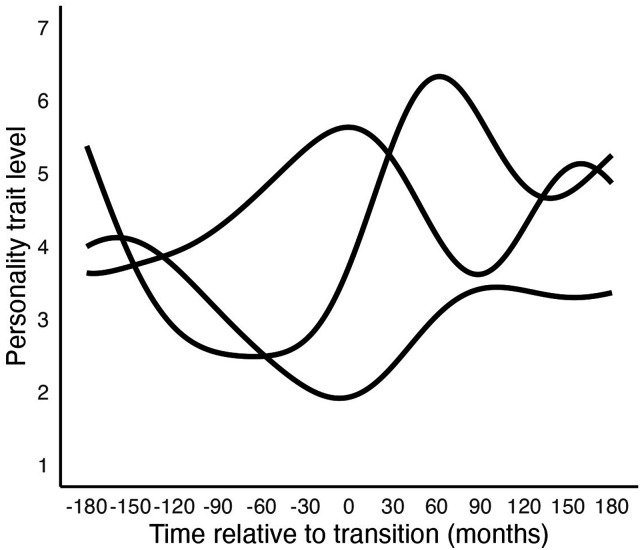
Illustration of how an EQ Kernel with the same magnitude and length scale can describe multiple shapes of Change. *Note.* All trajectories in this figure were generated with a length scale of 60 and a magnitude of 2.

While the parameters of the EQ kernel govern the rate and extent of change, they do not dictate its direction, allowing them to describe various shapes of change. This is illustrated in Figure 7, which shows diverse trajectories that are all described by the same magnitude and length scale. Consequently, to interpret the estimated trend, we need visualisation tools. A notable advantage of kernels in GPR is their ability to flexibly model any shape of change, while also providing parameters that describe the rate and extent of change. These parameters can be compared across groups or studies.

The length scale and magnitude parameters represent the average change across the trajectory. However, trajectories surrounding transitions are often characterised by more pronounced changes near the transition and relative stability further away from it. Therefore, the rate and extent of change are likely much larger near the transition than at greater distances. As a result, the average rate and extent of change across all time points may not accurately reflect such trajectories.

**Figure 8.**
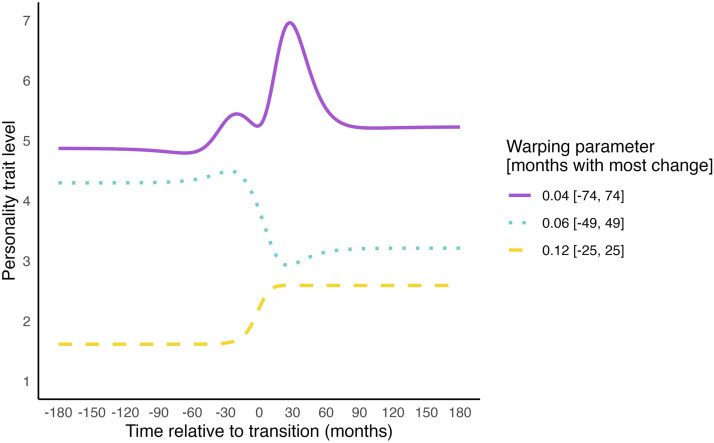
Illustration of the effect of adding input warping to the EQ Kernel. *Note.* This figure shows the effects of different warping parameter values for illustration purposes. Note that the warping parameter is estimated from the data during estimation.

To address this, we can add ‘input warping’ to the EQ kernel (Cheng et al., 2019; Snoek et al., 2014). Input warping transforms the time values (e.g. Time_a_ and Time_b_) into ‘warped’ time values (Time_a, warped_ and Time_b, warped_) before passing them into the kernel. This transformation ensures that most changes occur within a specific time window around the transition, and little change occurs further away from the transition. The breadth of this time window in which most change occurs is estimated empirically through a *warping parameter*. Combining the EQ kernel with input warping allows for the estimation of trajectories with both stable periods, and intensified change near transitions, as illustrated in Figure 8. Since input warping regulates wiggliness further away from the transition, it reduces the model’s susceptibility to capturing noise (overfitting). The warping parameter further allows us to interpret the time period during which the transition causes changes in the personality trait level.

To our knowledge, the only R package that supports GPR while accounting for individual differences is ‘lgpr’ (Timonen et al., 2021). This package allows the estimation of person-specific deviations for all parameters, similar to random effects in standard regression. This enables flexible estimation of unique change shapes for each individual. The model remains parsimonious, as it summarises these trajectories with a single deviation parameter (‘random effect estimate’) for each overall (‘fixed’) parameter. Additionally, the package supports testing the effects of moderators. A unique trajectory is estimated for each level of a moderator. Currently, only categorical moderators are supported, not continuous ones.

The ‘lgpr’ package uses a fully Bayesian modelling approach, as GPR was developed in this context. The Bayesian modelling requires defining a prior for each parameter before estimation. A prior is a probability distribution that reflects initial assumptions about a parameter. For example, if a personality trait is measured on a 10-point scale, the magnitude parameter (indicating the average deviation from the mean) cannot exceed 10. If we are unsure about the expected magnitude of change, we might use a non-informative prior. For instance, a normal distribution with a mean of 0 (i.e. no deviation from the mean) and a standard deviation (SD) of 2.5 would imply a 95% probability that the average deviation from the mean lies within ±5 (i.e. 2 SDs). If we know the personality trait level is unlikely to change more than 2 points, we could use a more informative (i.e. restrictive) prior with an SD of, for example, 1. Defining priors encourages researchers to consider what realistic ranges of parameter values might be before observing the data, and allows for the incorporation of knowledge from previous research, if available. If determining priors proves challenging, analyses can be conducted using various priors to ensure findings are robust, a process known as prior sensitivity analysis.

During model inference, the prior distributions are updated with the data, yielding posterior distributions for the parameters. That is, unlike frequentist inference which provides a single parameter estimate with an error margin, Bayesian inference produces a joint probability distribution for all parameters, representing the plausibility of various values after observing the data. This approach better accounts for parameter uncertainty.

A limitation of GPR is that it is computationally demanding. Estimation can be slow with large datasets. For example, modelling 1000 observations in total may take around 24 hours on a standard computer. Larger datasets may require more powerful computational resources, such as a computation server. Yet, efficient computation is an active area of development, and recent software improvements are making GPR more computationally feasible. Notably, the recently released ‘lgpr2’ package (Timonen & Lähdesmäki, 2025) offers substantially faster performance. However, documentation for this package is not yet available. While GPR may require some effort to implement, its flexibility and relative interpretability make it a promising approach for analysing personality change surrounding transitions.

### Summary

We discussed seven approaches to statistical modelling of non-linear personality change surrounding transitions. Table 1 summarises the approaches in terms of their main use cases, the insights they offer into aspects of change, and their key advantages, and limitations. Table 2 further details their data requirements, recommended R packages, and the potential for including random effects, multiple levels of nesting and moderators.

**Table 1.** Main use cases, insights, advantages, and limitations of approaches.

Approach	Use case: typical research question(s)	Use case: expected change shape	Insights into aspects of change	Key advantages	Main limitations
Linear regression on a transformed time variable	Quadratic: Does the trait increase or decrease surrounding the transition? When is the inflection point? Logarithmic: Does the trait increase or decrease after the transition? When does the change level off?	Simple predefined change shapes	Quadratic: Estimates rate of change; timing of peak/valley can be calculated Logarithmic: Estimates rate of change and indicates when changes level off	Easy to implement	Only simple shapes
Non-linear regression	For a Gaussian + logistic function: How much does the trait increase or decrease surrounding the transition? To what extent is the change sustained? When does the change occur? When is the inflection point?	Complex predefined shapes	For a Gaussian + logistic function: Estimates extent of change, timing of peak/valley, and stable levels; time period in which change occurs can be calculated	Flexibility and interpretability	Computationally demanding, potentially limiting the random effects structure
Piecewise regression	What changes occur before and after the transition?	Predefined segmented shapes, potentially discontinuous	Estimates extent of sudden shifts, and rate of change	Able to capture discontinuous (instantaneous) change	Dependence on correctness of predefined instantaneous changes
Changepoint analysis	When does the trend change, and how?	Data-driven shapes with two segments, potentially discontinuous	Estimates timing of sudden shift, and rate of change	Data-driven estimation of discontinuous (instantaneous) change	Flexibility of software implementations is limited
Continuous-time dynamic models	Does the transition cause a spike or dip in the trait? Is this spike or dip temporary or sustained? How do change estimates compare between samples using different measurement time intervals?	Shapes with a spike or dip at a known time point	Estimates rate of change, and extent of transition spike or dip. Note: Sizes of parameter values are only meaningful in comparison across groups or studies	Produces change estimates that are comparable across studies on different timescales	Cannot account for anticipatory transition effects
Generalised additive models	What is the shape of change?	Any continuous shape of change	Estimates shape of change; calculating the slopes across time points (as demonstrated in the example application) indicates the timing of changes	Flexible for identifying any continuous shape	Interpretation less clear, and potential underestimation of uncertainty
Gaussian process regression	What is the shape of change? How do estimates of the rate, extent, and timing of change compare between samples?	Any continuous shape of change	Estimates shape of change, extent of change, and rate of change. The time period in which change occurs can be calculated. Note: The rate of change parameter value is only meaningful in comparison across groups or studies	Flexible for identifying any continuous shape and offers parameters describing the change	Implementation can be complex due to the need for prior specification. Scalability is currently limited, although the recently released lgpr2 package is expected to significantly improve computational efficiency

**Table 2.** Data requirements, recommended R packages, and potential for including random effects, multiple nesting levels, and moderators across approaches.

Approach	Required time points	Sample size/power guidelines	R package recommendation		Random effects	Multiple nesting levels	Moderators
			Frequentist	Bayesian			
Linear regression on a transformed time variable	For logarithmic or square-root transformations: ≥2 For polynomials: The polynomial’s order +1	For sample size considerations see Raudenbush and Liu (2001). R package power calculations: simr (Green & MacLeod, 2016)	lme4 (Bates et al., 2024)	brms (Bürkner et al., 2024)	For all parameters	Possible	For all parameters
Non-linear regression	Complex shapes require more time points. As an indication, an exponential function should be possible from ∼4 time points per person (cf., Cudeck & Harring, 2007)	As an indication, an exponential function should be possible from ∼50 individuals (cf., Cudeck & Harring, 2007). Sample size determination is discussed in Kang and colleagues (2004), and Yan and Su (2006)	nlme (Pinheiro et al., 2024)	brms (Bürkner et al., 2024)	For some parameters. Potential convergence issues if added for all	Likely requires very large sample sizes	Requires very large sample sizes. Sample size determination is discussed in Yan and Su (2006)
Piecewise regression	The basic two-piece linear-linear model: ≥3 (two per segment, one shared at the intersection)	For power calculations, see Moerbeek (2022). R package power calculations: simr (Green & MacLeod, 2016)	lme4 (Bates et al., 2024)	brms (Bürkner et al., 2024)	For all parameters	Possible	For all parameters
Changepoint analysis	Should generally be possible with ∼5 per person (cf., Muggeo et al., 2014). Sufficient time points before and after the expected changepoint are required, as the changepoint cannot be estimated at the first or last two time points (McCormick et al., 2023)	Should generally be possible with 50 individuals (cf., Muggeo et al., 2014)	segmented (Muggeo, 2024) estimates only one changepoint, only straight-line segments, and only slope differences between segments (not sudden level shifts)	mcp (Lindeløv, 2024) estimates multiple changepoints (but see ‘random effects’ column)	Segmented: For changepoint, intercept, and slopes. However, it is often necessary to restrict the random effects correlations to zero for convergence mcp: For changepoint(s) only (not intercepts and slopes)	Not addressed in segmented’s, or mcp’s documentations	Segmented: For the changepoint, intercept, and slopes mcp: No moderators
Continuous-time dynamic models	Should generally be possible with ∼5 per person (cf., Haehner and Driver et al., 2025). Time point recommendations are discussed in Hecht and Zitzmann (2021)	Should generally be possible with 50 individuals (Driver & Voelkle, 2018a). Sample size recommendations are discussed in Hecht and Zitzmann (2021)	ctsem (Driver et al., 2017)	ctsem (Driver et al., 2017)	Frequentist: For intercepts only. Bayesian: For all parameters	Possible	Possible for all parameters by specifying a multiple group model
Generalised additive models	Generally ∼15 in total needed (Finch & Finch, 2018; Sullivan et al., 2015), possibly with each individual only sampled over part of the time range (McCormick et al., 2023)	Can be applied from 4 individuals if time points are sufficient (Sullivan et al., 2015)	mgcv (Wood, 2023). The estimated slopes across time points can be obtained using gratia (Simpson & Singmann, 2024)	brms (Bürkner et al., 2024)	For intercepts. If sufficient observations per person, random slopes (the linear trend through the wiggles), and curvatures (person-specific change shapes) are possible	Possible	Possible: A unique change shape will be estimated for each level
Gaussian process regression	Generally 6 per person needed (Timonen et al., 2021)	Should generally be possible from 16 individuals (cf., Timonen et al., 2021)	—	lgpr (Timonen & Johnson, 2023); and in the future, lgpr2 (Timonen & Lähdesmäki, 2025), once documentation becomes available	For all parameters. For each individual, a unique change shape is estimated	Not addressed in lgpr’s documentation	Categorical moderators only: A unique change shape will be estimated for each level

To summarise our main conclusions, linear regression on a transformed time variable is easy to implement and interpret, but limited in the range of change shapes it can model. In contrast, non-linear regression accommodates a wider range of shapes, including periods where personality traits remain stable. Simpler non-linear functions are straightforward to apply and interpret, but more complex functions may encounter convergence issues when including many random effects or moderators. Both linear and non-linear regression require that a mathematical function exists which approximates the expected shape of change. This is not required for piecewise regression, which allows modelling of custom shapes of change, including trajectories with sudden shifts. However, all three approaches – linear, non-linear and piecewise regression – rely on the researcher’s choice of function, reflecting their expectation of the shape of change. If this specification is incorrect, this can lead to misguided inferences. Changepoint analysis is somewhat less sensitive to misspecification, as it estimates a key characteristic of the shape of change empirically. While changepoint analysis is theoretically flexible for estimating various change shapes, current software is limited to two linear segments. CTDM is more flexible in estimating the post-transition non-linear shape of change from the data. However, it is the only model that, in its current application to studying transitions (Driver & Voelkle, 2018b) does not account for anticipatory effects − which may be a misspecification for many transitions (e.g. Denissen et al., 2019). Generalised additive models (GAMs) and Gaussian process regression are most flexible, estimating the shape of change entirely from the data. Therefore, they are least sensitive to misspecification. Note that these methods can only capture gradual, non-abrupt changes. GAMs are easier to fit but harder to interpret, while Gaussian process regression requires more effort to set up but can yield interpretable parameters on change extent, rate, and timing.

## Empirical example: Changes in life satisfaction surrounding widowhood

This section applies the approaches to an empirical example: life satisfaction changes surrounding the transition to widowhood. All analyses were conducted in R Statistical Software 4.4.2 (R Core Team, 2024). ggplot2 3.5.1 (Wickham, 2009) was used to visualise the results. The analyses can be reproduced using the code available at https://lisalevelt.nl/publications/modelling-nonlinearity/tutorial/.

### Data

Data from the Longitudinal Internet studies for the Social Sciences (LISS) panel were used. The LISS panel is a representative sample of Dutch adults, based on a true probability sample of households drawn from the population register. For further details on the LISS panel study, see Scherpenzeel and Das (2011). Data were used from the start of LISS in 2007 up until 2023. The LISS data can be accessed at https://statements.centerdata.nl/liss-panel-data-statement.

Panel members reported their marital status monthly, including an option for ‘widow or widower’. Changes in this status were used to indicate occurrences of widowhood. In addition, life satisfaction was measured annually with the 5-item Satisfaction With Life Scale (Diener et al., 1985). Participants responded on a 7-point scale, ranging from ‘strongly disagree’ to ‘strongly agree’. A mean of the items was computed where higher scores reflect higher levels of life satisfaction. In our sample, Cronbach’s alpha values ranged from .84 to .89 across all waves.

**Figure 9.**
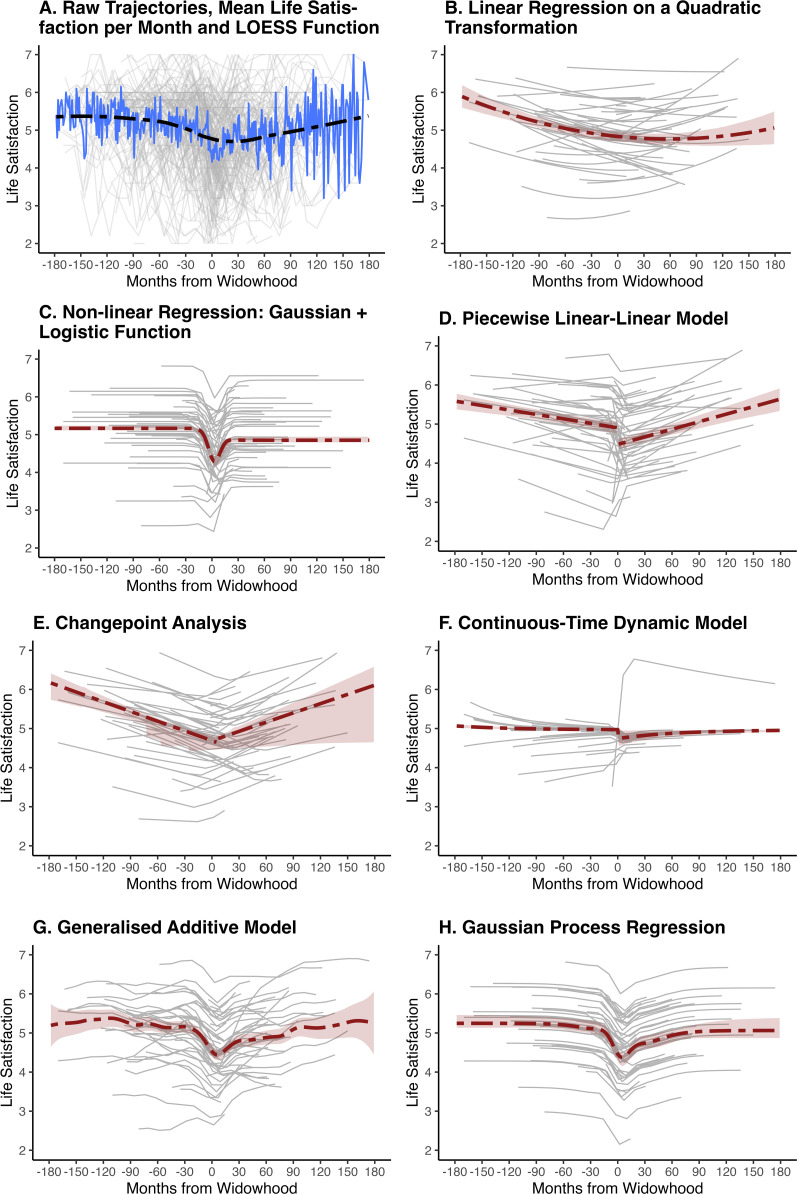
Average and person-specific life satisfaction change surrounding widowhood, as predicted by the models. *Note.* Panel A shows individual life satisfaction trajectories in grey (full sample, raw data), the mean life satisfaction per month in blue, and a LOESS function of life satisfaction in black (dashed). Panels B–H show model-estimated average trajectories in red (dash-dotted). Thin grey lines depict predicted trajectories for 50 randomly selected participants (same sample across all models). Red shaded areas indicate 95% confidence intervals for the average trajectories: calculated using 1000 bootstrap resamples for Models B−E; using the tidygam package 0.2.0 (Coretta, 2024) for Model G, and using 1000 posterior sample draws for Model F and H. Note that Panel F (continuous-time dynamic model) seems to show an outlier trajectory. A comparison of this individual’s predicted trajectory across all models is available at https://lisalevelt.nl/publications/modelling-nonlinearity/tutorial/CTDM.html#outlier-inspection, and shows that all other models produce similar predictions for this individual, but predict more variable trajectories for other individuals – masking this trajectory.

We included participants who became widowed during the study, and for whom at least one observation of life satisfaction was available both before and after the occurrence of widowhood. The final sample consisted of 2322 observations from *N* = 208 individuals (mean age at widowhood = 71.80, *SD* = 9.93; 82% female). On average, each person had 11 observations of life satisfaction (*SD* = 3.67). A time variable was created to indicate the timing of the life satisfaction observations in months relative to the occurrence of widowhood. Thus, Time = 0 marks the occurrence of widowhood, while negative values indicate the timing of observations before widowhood, and positive values indicate the timing of observations afterwards. Figure 9(a) illustrates the raw data pattern, showing individual life satisfaction trajectories (thin grey lines), the mean life satisfaction per month (thick blue line), and the LOESS-smoothed trend of life satisfaction (dashed black line).

### Analyses and results for each approach

#### Linear regression on a transformed time variable

To illustrate linear regression on a transformed time variable, we regressed life satisfaction on both a linear and a quadratic term of time. We chose a quadratic transformation because we expected an initial decrease in life satisfaction surrounding widowhood, followed by recovery (e.g. Infurna et al., 2017). To avoid multicollinearity arising from using two terms of time, we transformed the time variable to create linear and quadratic terms that were independent of each other, so-called orthogonal terms. The model was fit in lme4 1.1–35.5 (Bates et al., 2024), including random intercepts and slopes. Table 3 presents the results, and Figure 9(b) illustrates the average life satisfaction trajectory (red dash-dotted line) as predicted by the model. There was a significant negative linear effect of time, as well as a significant positive quadratic effect. This indicated that, surrounding widowhood, life satisfaction decreased initially, and later increased again. Life satisfaction was estimated to be lowest at 58 months after widowhood, at which point it was on average 4.77. The random effects estimates indicated substantial variability between individual-specific intercepts. The heterogeneity in the slopes was very small. There were no significant correlations among the random effects (i.e. the 95% confidence intervals contained zero).

**Table 3.** Results of quadratic model.

Parameter	*b*	95% CI	*p*	Random effects SD	95% CI
Intercept	4.95	[4.83, 5.07]	<.001	0.78	[0.70, 0.87]
Linear slope	−8.95	[−13.08, −4.76]	<.001	21.04	[17.36, 24.92]
Quadratic slope	5.94	[2.49, 9.37]	<.001	8.13	[1.05, 12.74]

#### Non-linear regression

To illustrate the application of non-linear regression, a combination of a Gaussian and a logistic function (as in Equation (3) and (4)) was estimated in nlme 3.1–166 (Pinheiro et al., 2024). Due to convergence issues, we were only able to include random effects for the baseline and step size parameters. Table 4 shows the results, and Figure 9(c) illustrates the life satisfaction trajectory as predicted by the model. The estimated baseline of life satisfaction was 5.17. Life satisfaction decreased by 0.88 points (on a 1–7 scale) on average surrounding widowhood, as indicated by the amplitude and half the step size.^4^ The centre parameter indicated that on average, life satisfaction was lowest at 2.51 months after widowhood, after which it started to increase to a new stable level, 0.32 points lower than before widowhood. The time span of this change was determined by solving for which time points the rate of change multiplied by the total change was greater than 0.01 (see note^5^ for calculations). This indicated that the change occurred roughly between 17 months before and 22 months after widowhood. The random effect standard deviation estimates were large, indicating large differences between individual-specific baselines and post-widowhood equilibria. The correlation of the random effects of the baseline and the step size was *r* = −.38 (95% CI [−.52, −.23]), indicating that individuals with higher pre-event life satisfaction experienced a smaller difference between their pre- and post-event stable levels.

**Table 4.** Results of the model combining a Gaussian and a logistic function.

Parameter	*b*	95% CI	*p*	Random effects SD	95% CI
Baseline	5.17	[5.04, 5.30]	<.001	0.86	[0.77, 0.96]
Step size	−0.32	[−0.47, −0.16]	<.001	0.93	[0.81, 1.07]
Amplitude	−0.72	[−0.83, −0.60]	<.001		
Width	6.54	[5.40, 7.68]	<.001		
Centre	2.51	[1.30, 3.72]	<.001		

#### Piecewise regression

To illustrate piecewise regression, we fit a two-piece linear-linear model in lme4 1.1–35.5 (Bates et al., 2024). We created the following time variables: postD, indicating the shift in the intercept after widowhood; preLin, indicating the rate of change before widowhood; and postLin, indicating the rate of change after widowhood. We standardised the preLin and postLin variables to avoid multicollinearity. We included random effects for all parameters.

Table 5 presents the results, and Figure 9(d) illustrates the life satisfaction trajectory as predicted by the model. The findings indicated a significant negative change in life satisfaction before widowhood, with a decrease of 0.05 points (on a 1–7 scale) per year on average.^6^ This change shifted to a positive 0.09 points per year after widowhood. Additionally, there was a significant sudden baseline shift, with the intercept decreasing by 0.42 points after widowhood.

**Table 5.** Results of the piecewise linear-linear model.

Parameter	*B*	95% CI	*p*	Random effects SD	95% CI
Intercept	5.17	[5.04, 5.30]	<.001	0.87	[0.78, 0.98]
PreLin	−0.16	[−0.23, −0.10]	<.001	0.24	[0.18, 0.31]
PostD	−0.42	[−0.56, −0.28]	<.001	0.78	[0.66, 0.91]
PostLin	0.23	[0.17, 0.30]	<.001	0.21	[0.13, 0.29]

Random effects showed substantial variability between individuals in intercepts and slopes. Two random effects were significantly correlated: the pre-widowhood intercept correlated positively with the pre-widowhood slope (*r* = 0.22, 95% CI [0.08, 0.56]), and negatively with the post-widowhood intercept-change (*r* = −0.51, 95% CI [−0.58, −0.27]). This indicated that individuals with higher pre-widowhood life satisfaction showed a steeper decline in life satisfaction before widowhood, but a smaller sudden decrease in life satisfaction after widowhood.

#### Changepoint analysis

To illustrate changepoint analysis, we fit a linear-linear model with the changepoint as unknown parameter in segmented 2.1–3 (Muggeo, 2024).^7^ Random effects were included for all parameters. Because of convergence issues, we had to restrict the correlation between the random effects to be zero. We used 0 as a starting value for the changepoint, and used 100 bootstrap resamples to mitigate potential sensitivity to starting values (Muggeo et al., 2014). Table 6 presents the results and Figure 9(E) illustrates the life satisfaction trajectory as predicted by the model. The change in life satisfaction was estimated to occur at 5.37 months after widowhood, at which point life satisfaction was estimated to be at 4.65 (on a 1–7 scale) on average. Before this changepoint, there was a significant decrease in life satisfaction of 0.09 points per year on average.^8^ Afterwards, life satisfaction showed a significant increase of 0.09 points per year on average. The estimated person-specific changepoints ranged from 5.24 to 5.53 months.

**Table 6.** Results of the changepoint analysis.

Parameter	*b*	95% CI	*p*	Random effects SD	95% CI
Overall intercept	4.68	[4.57, 4.81]	<.001	0.80	[0.72, 0.89]
Changepoint	5.37	[−3.80, 14.54]	^b^	1.59	[0.06, 45.35]
Slope before widowhood	−0.01	[−0.01, −0.01]	<.001	0.01	[0.00, 0.01]
Slope after widowhood^a^	0.01	[0.01, 0.01]	<.001	0.00	[0.00, 0.74]

*Note.* ^a^Changepoint analysis estimates the initial slope and the change in slope after the changepoint (as in equation (6)). For ease of interpretation, we report the actual post-changepoint slope and its significance, as is also provided by the segmented R package.

^b^A *p*-value for the changepoint location is not provided because its estimation is conditional on the presence of a slope change. In changepoint analysis, the significance of the slope change after the changepoint is estimated. Under the null hypothesis of no slope change (i.e. no changepoint), the location of the changepoint is undefined. Therefore, it is not statistically valid to simultaneously test for both the presence of a changepoint and its location. The presence of a changepoint can be tested separately using the Davies (1987) test or the pseudo-score test (Muggeo, 2016), both of which are implemented in the segmented R package.

#### Continuous-time dynamic model

To illustrate continuous-time dynamic modelling, we modelled the general change in life satisfaction with an initial level and an auto effect, using the R package ctsem 3.10.2 (Driver & Voelkle, 2018a). We specified a measurement model with the five observed life satisfaction items loading onto a latent life satisfaction factor. To estimate the impact of the widowhood transition, we created a transition time variable indicating the timing of the widowhood transition occurrence, and estimated a transition input effect and a transition auto effect.

We used the Bayesian estimation approach, allowing for random effects for all parameters and using the default weakly informative priors. To align with these priors, we coded the time variable in five-year intervals to reflect moderate change in life satisfaction over a time interval of 1 (cf., Denissen et al., 2019) and standardised life satisfaction scores (Driver & Voelkle, 2018a). To facilitate interpretation, the estimated initial life satisfaction level is presented on its original scale by multiplying it by the original standard deviation (SD) and adding the original mean. Its random effects SD is also rescaled by multiplying it by the original SD.

The results are presented in Table 7, and Figure 9(f) shows the life satisfaction trajectory as predicted by the model.^9^ The average initial life satisfaction level was estimated at 5.07. The negative auto effect indicates that life satisfaction tended to return to this level after fluctuations. The widowhood transition had a negative effect, and the negative widowhood auto effect indicates that this effect faded over time. The random effect estimates indicate that there was considerable between-person heterogeneity for all parameters. None of the random effects were significantly correlated.

**Table 7.** Results of the continuous-time dynamic model.

	Posterior mean	95% CI	Random effects SD	95% CI
Initial life satisfaction level^a^	5.07	[5.03, 5.11]	0.74	[0.65, 0.83]
Life satisfaction auto effect	−0.18	[−0.32, −0.08]	0.58	[0.36, 0.88]
Widowhood transition effect	−9.43	[−18.07, −1.02]	17.96	[4.88, 35.12]
Widowhood auto effect	−45.78	[−88.80, −3.09]	13.78	[3.14, 24.97]

*Note.* ^a^Life satisfaction was standardised to align with ctsem’s default priors, but the initial level mean and random effects SD estimates shown are in the original scale to facilitate interpretation.

#### Generalised additive model

To illustrate the generalised additive model, we fit a model with a fixed smooth function of time, and random (person-specific) smooth functions in mgcv 1.9–1 (Wood, 2023), using restricted maximum likelihood (REML) and thin-plate splines. A *k* of 333 was used – one fewer than the levels of the time variable, allowing a wiggle with each new level (cf., Doré & Bolger, 2018). Table 8 shows the results, and Figure 9(g) plots the trajectory as predicted by the model. The significant smooth of time indicated that life satisfaction changed over time. The effective degrees of freedom (EDF) of 17.13 suggested a non-linear pattern of change. Inspection of the slope changes over time, using the gratia package 0.9.2 (Simpson & Singmann, 2024), indicated that life satisfaction started to decrease at around 19 months before widowhood, started to increase at around 10 months after widowhood, and stabilised after approximately 24 months. Finally, the test for random effects supported person-specific curves, with the large EDF indicating considerable wiggliness in the individual-specific life satisfaction trajectories.

**Table 8.** Results of generalised additive model.

Parametric coefficient				
	*b*	SE	*t*	*p*
Intercept	4.98	0.06	83.79	<.001
Smooth terms				
	EDF	Ref.DF	*F*	*p*
Time	17.13	21.26	8.59	<.001
Time, individual	504.75	1711.00	3.16	<.001

*Note.* EDF = effective degrees of freedom; Ref.DF = reference degrees of freedom (used in computing the test statistic and *p*-values).

#### Gaussian process regression

To illustrate Gaussian process regression, we used the exponentiated quadratic kernel with input warping to model life satisfaction changes over time, allowing for potentially larger changes near the widowhood event. We used the R package lgpr 1.2.4 (Timonen et al., 2021), on a Windows Computation Server. The server had an Intel(R) Xeon(R) Gold 6258R CPU (24 processors) and 192 GB of RAM, running Windows Server 2022 Datacenter (version 21H2) on a 64-bit architecture. The estimation process took approximately 35 h. The lgpr package automatically standardises the response variable (in our case, life satisfaction) to have a mean of 0 and a standard deviation of 1 to enhance computational efficiency. However, to facilitate interpretation, the magnitude-estimate shown is on the original scale (through multiplication by the original standard deviation). The time variable was not standardised.

Wide, non-informative priors were used to permit a broad range of parameter values, only restricting them to be within ranges that align with the variables’ measurement units. Specifically, the prior for the magnitude parameter was set to expect average changes of 4 points or less, reflecting the maximum range of life satisfaction scores: Given that the mean life satisfaction score was approximately 5 on a scale from 1 to 7, the maximal deviation from this mean would be −4 points, corresponding to the lower bound of the scale. The priors for the length scale and warping parameters were chosen to accommodate both rapid and slow changes, as well as shorter and longer periods of change surrounding widowhood. For the residual variance, the prior only excluded negative values, permitting any plausible percentage. Further details on the priors can be found at https://lisalevelt.nl/publications/modelling-nonlinearity/tutorial/GPR.html#prior-specification.

In addition to estimating an overall (‘fixed’) effect of time, we initially estimated person-specific deviations for all parameters: magnitude, length scale, and warping parameter. However, this model showed poor fit. This was likely because the number of observations per individual was insufficient for this model. For this reason, we fit a simpler model with only static person-specific deviations from the overall effect of time, similar to random intercepts. This adjustment significantly improved model fit. We therefore interpret this final model.

The results are presented in Table 9, and Figure 9(h) illustrates the estimated change trajectory. On average, individuals experienced a decline in life satisfaction surrounding widowhood, followed by a nearly complete, but not total, recovery. The magnitude parameter for the overall time effect indicated a total drop of around one point on the 7-point scale.^10^ The warping parameter indicated that changes occurred within the time window of [−59, 59] months around the widowhood transition.^11^ In addition to the overall shared time effect, the average person-specific deviation from this effect was close to one point on the raw scale.

**Table 9.** Results of Gaussian process regression.

	Posterior mean	95% CI
Magnitude^a^	0.48	[0.22, 1.15]
Length scale	0.37	[0.18, 0.89]
Warping parameter	0.05	[0.03, 0.12]
Person-specific deviation^a^	0.81	[0.73, 0.90]

*Note.* ^a^Life Satisfaction was standardised for efficient estimation, but the magnitude and person-specific deviation estimates shown are in the original scale (through multiplication by the original standard deviation) to facilitate interpretation.

### Comparison of approaches

#### Interpretability

Applying the seven approaches to our empirical example revealed differences in both the quantity and nature of insights each approach generated. We will first consider which aspects of change could be interpreted from each model. In summary, the generalised additive model (GAM) and Gaussian process regression (GPR) allowed for interpretation of the shape of change. These models estimated the shape empirically, unlike the others, for which we prespecified it. Beyond shape, the GAM provided limited interpretability, mainly indicating when changes occurred. In contrast, non-linear regression generated many interpretable parameters. It captured the amount and timing of change, as well as stable levels before and after the transition, though the rate of change was more difficult to deduce. This rate was clear in both the piecewise and changepoint models. The piecewise model further quantified the extent of the sudden shift, and the changepoint analysis estimated the timing when the trajectory changed its course. However, neither of these approaches estimated when the changes began or ended on average, which the GPR did, alongside the extent and rate of change – though the interpretation of the rate of change was not very intuitive. The continuous-time dynamic model’s (CTDM) parameters for extent and rate of change indicated general change patterns but could not be interpreted in absolute terms. Finally, the quadratic model’s parameters were less easily interpretable than those of other approaches.

There were also notable qualitative differences between the insights generated by the approaches. Figure 9 highlights how the models differed in their estimated shapes of change and uncertainty levels. All approaches detected an initial decline in life satisfaction, followed by an increase. However, the piecewise and changepoint models, as we specified them (in line with common practice), suggested a complete return to pre-widowhood levels, while the other models indicated recovery was not fully complete (with CTDM suggesting very near, but not complete, recovery within the study window). The models provided similar, though slightly varied, estimates for the extent and rate of change, typically around 1 point on the 7-point scale, with a maximum change rate of 0.1 per year near widowhood. The CTDM plot indicated this model estimated the smallest change. In terms of timing, the non-linear regression and GAM estimated similar windows of change, from about 1.5 years before to 2 years after widowhood. The GPR, however, identified a broader change window, spanning 5 years before and after widowhood. Nevertheless, the GPR plot suggested that the largest changes occurred within the 2-year window, similar to the non-linear regression and GAM, with only minimal changes outside this period. Finally, estimates for when life satisfaction began to recover also varied widely, from 2.5 months post-widowhood in non-linear regression to 5 and 10 months in the changepoint analysis and GAM, and nearly 5 years post-widowhood in the quadratic model.

#### Performance

We evaluated the performance of the models based on four criteria. Firstly, we examined the balance between goodness of fit and model complexity, using the Bayesian Information Criterion (BIC), where a lower BIC indicates a better balance between model accuracy and complexity. Secondly, we assessed how well each model approximated the overall trend in the data by comparing the predicted average trajectory (based on the model’s fixed effects) with the actual average trajectory, which was calculated by averaging life satisfaction scores at each time point. To measure this, we computed three performance metrics using the caret 6.0–94 package (Kuhn, 2023): (1) *R*^*2*^, which indicates how closely the model’s predictions match the actual values, with higher values representing a better fit; (2) Mean Absolute Error (MAE), which reflects the average magnitude of the prediction errors, where lower values indicate more accurate predictions; and (3) Root Mean Squared Error (RMSE), which also reflects overall prediction error but penalises larger errors more heavily than MAE, with lower values signifying better predictive accuracy.

Thirdly, we evaluated how well each model captured individual trajectories by comparing the model’s random effects predictions with the actual person-specific life satisfaction trajectories, again using *R*^*2*^, MAE, and RMSE. Finally, we compared the extent to which each model’s average (fixed effects) trajectory generalised to new, unseen data using five-fold cross-validation. To do this, we randomly divided the full sample (*N* = 208) into five approximately equally sized participant groups. For each model, we estimated the average trajectory five times using four of the five groups (approximately 80% of the data; the training dataset, *N* ≥ 159), each time excluding a different group (approximately 20% the data; the test dataset). We then compared the estimated trajectory with the mean life satisfaction scores across time points in the excluded group. We computed the average *R*^*2*^, MAE, and RMSE across the five tests. Note that these values will be lower than those obtained from comparing the predicted average trajectory with the actual average trajectory for the full dataset. This is because we are now comparing predictions for one part of the data with actual values from another part not used in estimation. Nonetheless, these values provide insight into the relative generalisability of each model’s average trajectory to new, unseen data.

The performance evaluation results (Table 10) indicated that both the generalised additive model (GAM) and Gaussian process regression (GPR) captured the average trajectory very well, and generalised effectively to new data. The GAM performed excellently in capturing individual-specific trajectories, but it exhibited high model complexity (as indicated by the large BIC). The GPR had lower model complexity, using only four parameters to describe the change. However, it did not capture the individual-specific trajectories well, likely because the length scale and warping parameter were specified as being equal for all individuals, with only person-specific deviations estimated for the magnitude.

**Table 10.** Model performance indices.

Model	Balance model accuracy and complexity	Distance predicted average trajectory vs trajectory of mean values per time point			Distance predicted vs true individual-specific trajectories			Generalisability average trajectory			Computational time
	BIC	*R* ^*2*^	MAE	RMSE	*R* ^*2*^	MAE	RMSE	Average *R*^*2*^ *(SD)*	Average MAE *(SD)*	Average RMSE *(SD)*	
Quadratic regression	5590.19	0.14	0.33	0.43	0.70	0.46	0.61	0.06 *(0.04)*	0.63 *(0.07)*	0.83 *(0.10)*	<1 min
Non-linear regression	5359.14	0.28	0.29	0.39	0.73	0.43	0.58	0.10 *(0.02)*	0.59 *(0.07)*	0.74 *(0.00)*	<1 min
Piecewise regression	5356.08	0.27	0.29	0.39	0.77	0.40	0.53	0.10 *(0.07)*	0.58 *(0.08)*	0.76 *(0.13)*	<1 min
Changepoint analysis	5461.31	0.17	0.35	0.48	0.70	0.46	0.61	0.07 *(0.07)*	0.60 *(0.11)*	0.81 *(0.17)*	15 min
Continuous-time dynamic models	—^a^	0.24	0.33	0.43	0.27	0.81	1.00	0.09 *(0.03)*	0.60 *(0.07)*	0.78 *(0.10)*	<1 min
Generalised additive Model	7626.69	0.33	0.28	0.38	0.79	0.39	0.52	0.12 *(0.05)*	0.58 *(0.08)*	0.76 *(0.12)*	1 h
Gaussian process regression	—^a^	0.32	0.28	0.38	0.63	0.51	0.68	0.12 *(0.04)*	0.72 *(0.16)*	0.90 *(0.20)*	35 h^b^

*Note.* BIC = Bayesian Information Criterion (smaller is better); *R*^*2*^ = proportion variance explained (bigger is better); MAE = Mean Absolute Error (smaller is better); RMSE = Root Mean Squared Error (smaller is better).

^a^Not provided by the R packages used to estimate these models.

^b^On a computational server.

The piecewise and non-linear regression models performed relatively well overall, balancing goodness of fit, model complexity, and generalisability of the average trajectory to new data. Among these, the piecewise model was slightly better at capturing individual-specific trajectories. The CTDM and the changepoint analysis showed reasonable performance, but did not perform as well as the piecewise and non-linear regression models. CTDM captured the average trajectory quite good and generalised fairly well to unseen data, but was not effective in capturing the individual-specific trajectories. The changepoint analysis captured the individual-specific trajectories better, but performed less well on the average trajectory. This may be because the current software implementation does not support the explicit estimation of a sudden shift. The piecewise model suggested that such a shift was quite significant. Finally, the quadratic regression model showed the weakest performance in capturing the average trajectory, but outperformed CTDM in capturing the individual-specific trajectories.

## Discussion

To study non-linear personality changes surrounding transitions, we reviewed seven different statistical approaches. Each approach appeared suited to study different shapes of change, and each approach offered distinct strengths and limitations. If we have strong theoretical grounds to expect a specific shape of change, we can use researcher-specified functions. More specifically, if we expect a continuous (smooth) shape, we can use linear regression on a transformed time variable, or a non-linear regression model. If we expect a discontinuous shape (with instantaneous change), we can use piecewise regression and changepoint analysis. If we know when the change starts, but are unsure whether it is smooth or instantaneous, we can use continuous-time dynamic models (CTDMs). If we are unsure what timing and shape of change to expect, we can use data-driven approaches which flexibly capture any continuous trajectory: generalised additive models (GAMs) and Gaussian process regression (GPR). Improved changepoint analysis software enabling data-driven detection of multiple instantaneous changes may facilitate the modelling of known discontinuous shapes with more than two segments in the future.

Based on our findings, we offer a number of recommendations. A first recommendation is to choose a statistical approach based on *what we know* (or do not know) about the shape of change. Researcher-specified approaches can produce misleading results if the assumed shape is incorrect. If the theoretical framework lacks clarity or robustness regarding how the personality trait change unfolds over time, adopting empirically driven approaches is essential (Hopwood et al., 2022; Wright & Jackson, 2024). In such a case, allowing the data to reveal the trajectory of personality change as it is, instead of constraining it to fit a potentially mismatched model, is key to developing and refining theories (Adolph et al., 2008; Van Lissa, 2023; Wright & Jackson, 2024).

A second recommendation is to more explicitly consider *what we want to know* when choosing a modelling approach. Data-driven approaches are often seen as having higher predictive value at a cost of lower interpretability, while researcher-specified approaches are seen as more informative but less predictive (Yarkoni & Westfall, 2017). Our findings partially support this distinction. Indeed, the data-driven approaches showed the best performance in the empirical example, in terms of accounting for the average trajectory, the person-specific trajectories, and generalisability to new data. In contrast, the researcher-specified approaches, except for the CTDM and the quadratic regression, quantified the most change aspects in a way that allowed for interpretation. However, contrary to the sometimes negative view that more data-driven techniques are less interpretable (Stachl et al., 2020), we have demonstrated that GAMs allow for interpreting the shape and timing of personality change, and GPR offers estimates of the extent and rate of change which can be compared across groups. Furthermore, this paper highlights that not all researcher-specified approaches offer the same insights. For example, non-linear or piecewise regression was useful for quantifying the extent of change, but changepoint analysis was better suited for identifying when the trajectory shifts. When choosing a modelling approach, it is therefore important to consider what aspects of the personality change we are interested in: its shape, timing, extent, or another aspect? By aligning methods with research objectives, we can more effectively address our questions.

### Insights from the empirical example

The application of the modelling approaches to the empirical example of changes in life satisfaction around widowhood revealed notable qualitative differences in results. Most strikingly, the piecewise and changepoint models suggested that life satisfaction, after an initial decline, fully recovered to pre-widowhood levels in the long run. This would support set-point theories (Headey, 2006; Lykken & Tellegen, 1996; Ormel et al., 2017), which posit that personality traits fluctuate around person-specific set points, and while life transitions may cause temporary changes, individuals generally adapt and return to their person-typical levels. However, the other models indicated incomplete recovery, consistent with studies showing that personality can undergo lasting changes surrounding life transitions (e.g. Bühler et al., 2023; Denissen et al., 2019; Lucas, 2007). It is likely that we did not find partial recovery in our piecewise and changepoint models because of how we specified them: allowing only one trend after the transition, as is common practice in studies on personality changes surrounding transitions. Such an approach permits the estimation of either no recovery or full recovery over the long term, but not partial recovery. This underscores how modelling choices can shape findings, which may have theoretical implications.

Given that the choice of statistical approach can influence results, a third recommendation is to critically consider the (sometimes implicit) assumptions inherent to our chosen method when interpreting results. For instance, we should not interpret the pre-widowhood decline in life satisfaction observed in the piecewise model as the total decline surrounding widowhood, as is sometimes done. This is because the decline may continue for some time after the widowhood occurrence, resulting in a larger overall decline, which the piecewise model’s specification cannot fully capture.

A final recommendation is to conduct robustness checks. This includes testing alternative model specifications, such as exploring different transformations of time, or adding segments with varying forms to piecewise models. Cross-validation, where the dataset is repeatedly split into training and test sets to evaluate model performance, is also essential to provide a robust measure of model performance and reliability, although it is only rarely conducted. Cross-validation does require a sufficiently large sample to allow for splitting, and the percentage of the data used for training should be large enough to reliably estimate model parameters. Additionally, the assumptions of researcher-specified models can be validated empirically using data-driven techniques. For example, shapes of change identified through GAM or GPR can inform the specification of a researcher-defined model to interpret specific aspects of change, or changepoint modelling can identify where the trajectory typically shifts, guiding segment specification in piecewise models. Such practices can improve the reliability of findings and strengthen their theoretical interpretations.

While it might sound hypocritical since we did not preregister our analyses ourselves, we emphasise the importance of preregistering analysis plans and robustness checks to ensure transparency and reduce the risk of data-driven bias. Anticipating all necessary specifications and potential issues during model fitting can be difficult. Through this paper, we hope to support preregistration practices by providing a guide for informed modelling decisions among approaches, and an overview of considerations and challenges per approach. Additionally, the accompanying website illustrates the modelling steps for each method, helping researchers prepare for their analyses.

### Alternative approaches

Our choice of approaches stems from their strengths in capturing non-linear population-level change. However, other approaches are also commonly used in personality research. We briefly consider three alternatives. Firstly, fixed-effects models (for introductions, see Brüderl & Ludwig, 2014; Hill et al., 2020), allow for examining changes surrounding transitions without assuming a specific functional form. We did not include them because, for studying overall population change, they require consistent measurement timings across individuals or the grouping of measurements into broader categories, which reduces temporal precision – an important limitation when studying non-linear change (McCormick et al., 2023). Nevertheless, fixed-effects models are useful when the primary aim is to isolate within-person changes, or when creating a control group is challenging (e.g. see Haehner and Krämer et al., 2025). Secondly, we did not focus on discrete-time structural equation models (SEM), which also require consistent measurement timings across individuals. However, note that all researcher-specified approaches we considered can be implemented within the SEM framework. SEM also offers the latent basis model (McArdle, 2009), which allows the shape of change to be freely estimated, similar to the data-driven approaches. Implementing these models within the SEM framework enables the inclusion of latent variables and to extend the models to study correlated changes across multiple variables over time. If the primary objective is studying such interrelationships rather than identifying the precise non-linear change over time, we recommend existing introductions to these methods within the SEM framework (e.g. Grimm et al., 2011; Harring et al., 2021). Finally, the Individual Network Invariance Test (INIT; Hoekstra et al., 2024) can be used to assess whether an individual’s structure of associations among multiple variables (e.g. multiple facets of conscientiousness; c.f., Borsboom et al., 2021) changes across two time periods, such as before and after a transition. However, because INIT does not capture the (potentially non-linear) trajectory of change, it is not included in this paper.

### Limitations

This study has some limitations. As our aim was to assess the utility of the approaches in a real-world context, the use of real data was most appropriate. We therefore did not simulate data with known change patterns, meaning we could not compare the fitted trajectories with the true trajectories. The performance of some of the models has been evaluated in simulation studies before, however, such as by Fine and Grimm (2021) and Sciarra (2023). Moreover, for the data-driven techniques, running a substantial number of simulations under multiple conditions – such as varying change shapes, effect sizes, and error structures – was infeasible due to their high computational demands, even with access to a computational server. Our performance evaluation findings are thus based on a case study reflecting a specific change pattern, relying on particular versions of each approach (e.g. a quadratic rather than cubic transformation of time). This limits the generalisability of our findings to other change patterns or alternative specifications of the approaches. However, we chose a typical example of personality change surrounding transitions, characterised by both temporary and discontinuous aspects, and aimed to specify the approaches as commonly done. These choices provide a meaningful context for comparison. While our study does not provide a comprehensive statistical evaluation, its value lies in offering a hands-on introduction and conceptual and illustrative comparison of different modelling approaches.

### Conclusion

We hope this paper adds new statistical approaches to the reader’s toolbox and offers an overview to guide the selection of the appropriate methods for studying non-linear changes. This is important for the study of personality change surrounding transitions, as incorrect modelling approaches can lead to its over- or, more likely, underestimation (Wright & Jackson, 2024). In fact, change over time is virtually always non-linear, even outside of transitions, making this paper relevant not only to psychology but also to longitudinal research in fields such as medicine, sociology, and economics. We hope this paper inspires new research on non-linear personality change surrounding transitions, and that with studies incorporating our recommendations, a fuller understanding of these changes can be achieved.

## Notes

1. Other functions can also capture this shape and could be used as well. We have chosen the Gaussian function because it is well-known.

2. Other sigmoidal functions could also be used, such as a Gompertz function.

3. Changepoint analysis is also implemented in Mplus (Muthén and Muthén, 1998) as described by Kohli and Harring (2013), and in SAS (Institute Inc, 2008) as described by Kohli and Colleagues (2019). We did not test these implementations, but the sources indicate they do not offer additional features beyond those provided by the R packages we considered.

4. When using the same centre and width parameters in both the Gaussian function part and the logistic function part, the height of the peak/valley can be found by adding half of the step size to the amplitude, as the logistic function reaches halfway its step at its centre.

5.
$total\;change\times \exp\left(-\frac{{\left(\text{Time}-centre\right)}^{2}}{2\times {width}^{2}}\right)>0.01$ ,
$0.88\times \exp\left(-\frac{{\left(\text{Time}-2.51\right)}^{2}}{2\times 6.54^{2}}\right)>0.01$ , Time ≈ −17, and Time ≈ 22.

6. The yearly change coefficients were calculated by first converting the standardised monthly coefficients to unstandardised ones using $b=B*\left(\frac{sd\left(\text{Life}\;\text{satisfaction}\right)}{sd\left(\text{preLin}\right)}\right)$ , and then multiplying by 12.

7. We also attempted to fit a Bayesian changepoint model using the R package mcp (Lindeløv, 2024), but it failed to converge. This was likely due to the inability of the package to account for individual differences in intercepts and slopes, which, as found in the other approaches, are substantial.

8. The yearly change coefficients were calculated by multiplying the monthly change coefficients by 12.

9. Note that ctsem provides standard plotting functions for estimated individual trajectories, which use the observed data to update the model predictions via a Kalman filter. However, to ensure comparability with the other models – which do not use such updates – we manually plotted trajectories based solely on the model equations.

10. The magnitude parameter indicates the deviation from the mean of the trajectory. As shown in Figure 9(h), the mean of the trajectory lies approximately halfway through the initial drop in life satisfaction. Therefore, the estimated total decline in life satisfaction is around twice the deviation indicated by the magnitude parameter.

11. The change window was determined by inserting the mean warping parameter estimate into the warping function and solving for the time values at which the warped inputs approached |1|, that is: 
$|0.9|=2\times \left(\frac{1}{1+\exp\left(-warping\;parameter\;\cdot \text{Time}\right)}-\frac{1}{2}\right)$ .
